# Associations between brain inflammatory profiles and human neuropathology are altered based on apolipoprotein E ε4 genotype

**DOI:** 10.1038/s41598-020-59869-5

**Published:** 2020-02-19

**Authors:** Jacob S. Friedberg, Nurgul Aytan, Jonathan D. Cherry, Weiming Xia, Oliver J. Standring, Victor E. Alvarez, Raymond Nicks, Sarah Svirsky, Gaoyuan Meng, Gyungah Jun, Hoon Ryu, Rhoda Au, Thor D. Stein

**Affiliations:** 1 Boston University Alzheimer’s Disease and CTE Center, Boston University School of Medicine, Boston, MA 02118 USA; 2 Department of Neurology, Boston University School of Medicine, Boston, MA 20118 USA; 3 VA Boston Healthcare System, Boston, MA 02130 USA; 4 Department of Veterans Affairs Medical Center, Bedford, MA 01730 USA; 5 Department of Pathology and Laboratory Medicine, Boston University School of Medicine, Boston, MA 02118 USA; 6 Department of Medicine (Biomedical Genetics), Boston University School of Medicine, Boston, MA 02118 USA; 7 Department of Biostatistics, Boston University School of Medicine, Boston, MA 02118 USA; 8 Framingham Heart Study, Boston University School of Medicine, Boston, MA USA; 9 Department of Epidemiology, Boston University School of Public Health, Boston, MA USA

**Keywords:** Alzheimer's disease, Neurodegeneration

## Abstract

Alzheimer disease (AD) is a chronic neurodegenerative disease with a multitude of contributing genetic factors, many of which are related to inflammation. The *apolipoprotein E (APOE) ε*4 *allele* is the most common genetic risk factor for AD and is related to a pro-inflammatory state. To test the hypothesis that microglia and AD-implicated cytokines were differentially associated with AD pathology based on the presence of *APOE* ε4, we examined the dorsolateral frontal cortex from deceased participants within a community-based aging cohort (*n* = 154). Cellular density of Iba1, a marker of microglia, was positively associated with tau pathology only in *APOE* ε4 positive participants (*p* = 0.001). The cytokines IL-10, IL-13, IL-4, and IL-1α were negatively associated with tau pathology, independent of Aβ_1–42_ levels, only in *APOE* ε4 negative participants. Overall, the association of mostly anti-inflammatory cytokines with less tau pathology suggests a protective effect in *APOE* ε4 negative participants. These associations are largely absent in the presence of *APOE* ε4 where tau pathology was significantly associated with increased microglial cell density. Taken together, these results suggest that *APOE* ε4 mediates an altered inflammatory response and increased tau pathology independent of Aβ_1–42_ pathology.

## Introduction

Alzheimer’s disease (AD), the leading cause of dementia, is a progressive neurodegenerative disorder with both genetic and environmental risk factors. Many of the genes associated with AD have small effects and are associated with immunological and inflammatory pathways in the brain^1,2^. In contrast, the *apolipoprotein E (APOE) ε*4 allele is associated with a dramatic increase in the risk of developing AD and a younger age of symptom onset^3,4^. APOE may contribute to the development of AD through a variety of mechanisms, including increased beta-amyloid (Aβ) levels as well as an altered neuroinflammatory state.

Studies examining human post-mortem brain tissue found an increased number of microglia within AD patients with *APOE* ε4^5,6^. Minett *et al*.^7^ found that increased microglia levels were negatively associated with AD pathology in non-demented individuals while the opposite was observed in demented individuals. Furthermore, *APOE ε*4 was associated with increased microglial activation and worse tau pathology and clinical outcomes^7^. In addition, peripheral low-grade inflammation was associated with risk of AD in *APOE ε*4, but not *ε*2 or *ε*3 carriers^8^. These studies suggest the *APOE ε*4 allele may lead to AD pathology through an altered inflammatory state. However, the precise role of *APOE* ε4 in modulating the relationship between microglia and inflammatory cytokines with the development of AD pathologies in the human brain is largely unknown.

Multiple cytokines have been implicated in AD pathogenesis. These include both the generally pro-inflammatory cytokines IL-1α and β, TNF- α, and IFN-γ as well as the anti-inflammatory IL-4, IL-10, and IL-13. Pro-inflammatory cytokines may initially reduce pathology by clearing amyloid and tau but may become toxic over time. Anti-inflammatory cytokines have been associated with decreased levels of pro-inflammatory cytokines and increased neurogenesis in mouse models of AD^9–12^. A recent study by Taipa *et al*. found that a collection of both pro-inflammatory and anti-inflammatory cytokines were correlated with increased cognition and less cognitive decline in AD patients after one year, suggesting that a balance of cytokines is important for maintaining a less pathologic immune profile^13^. The effective loss of this balance and increased inflammation may contribute to the progression of AD pathology^13,14^. Overall, cytokines released from microglia may help determine the prevailing inflammatory state within the brain^15^. However, opposing results have been reported on the impact of cytokines on AD pathology and symptoms based primarily on mouse models and human blood and CSF^15–20^. Few studies have examined these relationships in human brain tissue and in relationship to APOE ε4 status. Thus, we aim to elucidate the effect of pro-inflammatory and anti-inflammatory cytokines on AD pathology within frontal cortical tissue in relationship to *APOE* genotype. Overall, the role of microglia in attenuating or facilitating the development of AD pathologies may be mediated by the specific factors they release and the balance between pro-inflammatory and anti-inflammatory cytokines.

Here we examine the dorsolateral frontal cortex, a region where Aβ develops early and tau pathology develops late, in a community-based aging cohort in order to study the age dependent relationships between known inflammatory cytokines and AD pathologies. We sought to test the hypothesis that the presence of the *APOE* ε4 allele alters the interaction between microglia and associated cytokines with AD pathology.

## Methods

In the present study, we examined 154 autopsy participants from the Framingham Heart Study (FHS). The FHS is a community-based cohort that longitudinally tracks participants and their offspring using previously published selection criteria and protocols^21^. Participants who agreed to brain donation were enrolled and tissue was collected after death with informed consent from the next of kin. Methods were carried out in accordance with and approved by the institutional review boards from both the Boston University Medical Center and the Edith Nourse Rogers Memorial Veterans Hospital, Bedford, MA.

### Pathological assessment

Neuropathological assessment was performed following procedures and criteria previously established by the Department of Veterans Affairs-Boston University brain bank^22,23^. Alzheimer disease was diagnosed based on the National Institute of Aging Reagan criteria. Alzheimer disease staging was performed according to the Braak and Braak staging for neurofibrillary tangles^24^ and the Consortium to Establish a Registry for Alzheimer Disease (CERAD) semi-quantitative criteria for neuritic plaques^25^. Neuritic plaques were defined as plaques with argyrophilic dystrophic neurites, with or without dense amyloid cores^26^.

### Immunohistochemistry

Tissue was fixed in periodate-lysine-paraformaldehyde, tissue blocks were paraffin-embedded, and sections were cut at 10 µm for immunohistochemistry. Antigen retrieval for α-synuclein and β-amyloid was performed with formic acid treatment for two minutes. Sections were incubated overnight at 4 °C with antibodies to phosphorylated PHF-tau (AT8; Pierce Endogen, Rockford IL; 1:2000), ionized calcium binding adaptor molecule 1 (Iba1) (Wako, 1:500), and CD68 (Vector, 1:500). Sections were washed three times with phosphate-buffered saline (PBS; pH 7.4), and subsequently treated with biotinylated secondary antibody and labeled with a 3-amino-9-ethylcarbazol HRP substrate kit (Vector Laboratories, Burlingame, CA). The sections were then counterstained with Gill’s Hematoxylin (Vector Laboratories H-3401, Burlingame, CA) and subsequently cover slipped using Permount mounting medium.

### Immunoassay measurement

Frozen tissue from the dorsolateral frontal cortex was weighed and placed on dry ice. Freshly prepared, ice cold 5 M Guanidine Hydrochloride in Tris-buffered saline (20 mM Tris-HCl, 150 mM NaCl, pH 7.4) containing 1:100 Halt protease inhibitor cocktail (Thermo Fischer Scientific, Waltham, MA) and 1:100 Phosphatase inhibitor cocktail 2 & 3 (Sigma-Aldrich, St. Louis, MO) was added to the brain tissue at 5:1 (5 M Guanidine Hydrochloride volume (ml):brain wet weight (g)) and homogenized with Qiagen Tissue Lyser LT at 50 Hz for five min. The homogenate was then mixed (regular rocker) overnight at room temperature. The lysate was diluted with 1% Blocker A (Meso Scale Discovery (MSD), Rockville, Maryland, #R93BA-4) in wash buffer according to specific immunoassays: 1:300 for total tau and pTau231 (MSD #K15121D-2) and 1:4000 for beta-amyloids 1–40 and 1–42 (MSD #K15200E-2). Samples were subsequently centrifuged at 17,000 g and 4 °C for 15 minutes, after which the supernatant was applied to the immunoassays.

Levels of AD related cytokines including IFN-γ, IL-1β, IL-4, IL-10, IL-13, TNF-α, and IL-1α, were determined using the MSD proinflammatory panel 1 and cytokine panel 1. Ice cold RIPA buffer (Thermo Scientific, #89901) was added to the brain tissue at 5:1 and homogenized with Qiagen Tissue Lyser LT at 50 Hz for five min. The homogenate was centrifuged at 17,000 g and 4 °C for 15 minutes, then the supernatant was aliquoted for further use. Cytokine measurements were preformed according to MSD protocol using sandwich immunoassay techniques (MSD# K15210G). Brain homogenate was diluted with MSD diluent 21 and incubated for 2 hours with shaking at 500 rpm and subsequently washed three times with PBS + 0.05% Tween-20. Cytokine antibody was then added and the mixture was incubated for 2 hours with shaking at 500 rpm, and again subsequently washed three times with PBS + 0.05% Tween-20. 2X Read buffer T (MSD #R92TC-3) was added prior to analysis. Sulfo-tag conjugated anti-mouse secondary antibody was used for signal detection by the MSD platform, and an MSD SECTOR Imager 2400 was used to measure analyte levels.

### Microscopic analysis of Tau and Microglia density

Tissue blocks from the dorsolateral frontal cortex were embedded in paraffin and cut at 10 μm (AT8) or 20 μm (Iba1 and CD68). Slides immunostained for tau (AT8), microglia overall (Iba1), and activated microglia (CD68) were scanned at 20× magnification with a Leica Aperio Scanscope (Leica Biosystems, Buffalo Grove, IL) as previously described^27^. Briefly, using ImageScope (Leica Biosystems, Buffalo Grove, IL), the gray matter was highlighted from the pia to the boundary between the white and gray matters. Leica’s image analysis and automated counting software (Aperio nuclear algorithm, Version 9) was calibrated for shape, size, and staining intensity to detect AT8-positive NFTs, Iba1-positive cells, and CD68-positive cells within the region of interest. Counts were normalized to the area measured and are presented as density within the analyzed region.

### *APOE* genotyping

*APOE* genotype was determined from DNA extracted from participant brain tissue using single nucleotide polymorphism (National Center for Biotechnology Information SNPs rs429358 and rs7412) as previously described^28^. Participants were divided into *APOE* positive, which included genotypes ε2/ε4 (n = 2), ε3/ε4 (n = 30), and ε4/ε4 (n = 3), and *APOE* negative, including ε2/ε2 (n = 1), ε2/ε3 (n = 10), and ε3/ε3 (n = 98).

### Statistical analysis

Statistical analysis was performed with SPSS version 25.0 (IBM inc., Armonk, NY) and Prism v8 (Graphpad Software, La Jolla, CA). IHC cellular density data – AT8, Iba1, CD68 – was analyzed as positive pixel count per mm^2^.

A total of 154 cases with complete data were included in the study. Levels of AT8, IL-10, IL-13, IL-4, IL-1β, IL-1α, and IFN-γ were normalized by logarithmic correction. Aβ measures were not normally distributed after log or square root corrections and therefore divided into quartiles and used as an ordinal variable. The raw data for Iba1, CD68, and TNF-α were normally distributed and needed no correction. Outliers were defined by values 3 times the interquartile range away from the upper and lower limits of the interquartile range for each measure and cases were removed from the dataset. In order to allow comparison, participants were divided into groups with and without an *APOE* ε4 allele.

To assess the variance between *APOE* ε4 carrier and non-carrier groups with respect to demographic and neuropathological characteristics we applied a chi-square test for proportions and independent samples T-tests. We used separate ordinal logistic regression models to estimate the relative odds of a one quartile increase in Aβ levels for a unit increase in microglial cell densities as well as cytokine levels adjusting for age and sex. Multiple linear regressions were used to test associations between microglial cell densities and tau pathology density and between cytokines and tau pathology adjusting for age, sex, and Aβ_1–42_. Analyses for each cytokine were run separately to avoid collinearity issues. Multiple comparisons were accounted for using Bonferroni correction. Binary logistic regressions were used to assess the relationship of drivers with dementia.

## Results

Of the 154 participants from the FHS cohort analyzed there were 119 *APOE* ε4 negative and 35 were *APOE* ε4 positive. These two groups varied significantly by frequency of AD, cerebral amyloid angiopathy (CAA), and dementia such that the *APOE* ε4 carriers group had a higher prevalence. The mean level of tau pathology (AT8 density) was higher in *APOE* ε4 positive participants than *APOE* ε4 negative (Table 1). IL-13 and IL-4 had higher levels on average in *APOE* ε4 negative than *APOE* ε4 positive participants (Table 2). To test whether there was an effect of an interaction between *APOE* ε4 genotype and sex on tau pathology, a two-step linear regression adjusting for sex, *APOE* ε4, and sex-*APOE* ε4 interaction term was performed. The presence of *APOE* ε4 was significantly related to tau pathology (p = 0.007) independent of sex but the interaction term was not (p = 0.947). This suggests that the effect of *APOE* ε4 on tau pathology is independent of sex.

**Table 1:** Demographic and neuropathological characteristics of cohort.

	APOE ε4 negative	APOE ε4 positive	*p*-value
**Demographics**			
Sample size (n)	119	35	—
Age at Deaths (yrs)^b^	87.9 (0.843)	85.9 (1.63)	0.657
Sex (% male)^a^	43.7%	45.7%	0.788
Dementia^a^	46.2%	65.7%	**0.043**
**Pathology**^**a**^			
AD	28.6%	60.0%	**0.001**
CTE	0.84%	2.86%	0.354
LBD	27.7%	37.1%	0.285
Neocortical LBD	8.40%	8.57%	0.975
FTLD	11.8%	11.4%	0.957
Remote Cortical Microinfarct^#^	40.4%	40.0%	0.969
Large Infarcts	17.6%	22.9%	0.488
Arteriolosclerosis	86.6%	91.4%	0.441
Atherosclerosis	75.6%	77.1%	0.854
CAA	75.6%	91.4%	**0.043**
**IHC cellular density (cells/mm**^**2**^**)**^**b**^			
AT8	12466 (4614)	29741 (9678)	**0.028**
Iba1	174.96 (4.04)	181.98 (8.47)	0.448
CD68	166.85 (5.72)	187.25 (12.69)	0.162
**Immunoassay (pg/mL)**^**b**^			
Aβ_1–42_	864 (74)	1330 (180)	**0.018**
Aβ_1–40_	177 (53)	653 (156)	**0.007**

LBD: Lewy body disease; AD: Alzheimer’s disease; CTE: Chronic Traumatic Encephalopathy; FTLD: Frontotemporal Lobar Degeneration; CAA: Cerebral Amyloid Angiopathy; Pathology prevalence represent the presence of pathology not the primary diagnosis; Control cases were defined as those lacking AD, CTE, LBD, or FTLD pathology. ^a^χ^2^ test for proportions between ε4 allele absent and present groups; values presented as a percentage. ^b^Independent sample t-test for equality of means; values presented as mean (S.E.M). ^#^Not all cases had remote cortical microinfarct data available (N = 144).

**Table 2:** Levels of AD-related cytokines by *APOE* genotype.

	ε4 Negative	ε4 Positive	*p*-value^a^
IL-10	0.240 (0.007)	0.244 (0.0153)	0.119
IL-13	4.02 (0.116)	3.47 (0.135)	**0.003**
IL-4	0.302 (0.0135)	0.261 (0.0204)	**0.004**
TNF-α	0.517 (0.0161)	0.517 (0.0236)	0.211
IL-1β	4.38 (0.734)	4.19 (0.716)	0.406
IL-1α	1.46 (0.0990)	1.34 (0.104)	0.207
IFN-γ	1.54 (0.0452)	1.65 (0.0941)	0.242

Measures are presented as mean concentration (S.E.M) pg/mL; ^a^independent sample t-test for equality of means.

### Associations between microglia and AD pathological markers

**Figure 1:**
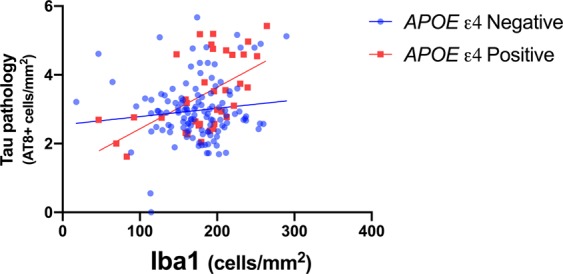
*APOE* ε4 allele alters the relationship between Iba1 and tau pathology. Scatter plots of Iba1 versus tau pathology (AT8 immunohistochemistry) are shown. The FHS cohort was divided into *APOE* ε4 positive and negative groups (red circles and blue squares respectively). Linear regressions demonstrated that Iba1 displayed significant positive relationship with tau pathology only in *APOE* ε4 carriers (B = 0.012, p =  <0.001, 95% CI = 0.006–0.019).

To test the hypothesis that the presence of the *APOE* ε4 allele altered the relationship between microglia and amyloid and tau pathology we examined these associations separately in participants with and without the *APOE* ε4 allele. Scatter plots of *APOE* ε4 positive and negative groups demonstrate significantly different associations between microglia cell density and AD pathology such that Iba1 was positively associated with the log of AT8 in *APOE* ε4 positive participants, but displayed no significant association in *APOE* ε4 negative participants (Fig. 1). In order to adjust for the effects of other potential drivers of pathology, we performed multiple regression analyses. A multiple linear regression showed that Iba1 cell density was significantly associated with greater AT8 positive tau pathology density (B = 0.013, *p* = 0.001) in *APOE* ε4 positive, but not *APOE* ε4 negative, participants adjusting for age, sex, and Aβ_1–42_. Iba1 cell density was not significantly associated with Aβ_1–40_ or Aβ_1–42_ via logistic ordinal regressions controlling for age and sex. Ordinal logistic and multiple linear regression analysis of amyloid and tau pathology with CD68 did not exhibit any significant relationships (Table 3). There is a clear difference in the relationship of Iba1, but not CD68, with tau pathology depending on the presence of the *APOE* ε4 allele. These results show that within *APOE* ε4 positive, but not *APOE* ε4 negative participants, microglial density is associated with increased tau pathology.

**Table 3:** Associations of tau and Aβ pathologies with total (Iba1) and activated (CD68) microglia in APOE ε4 negative and positive participants.

**AT8** ^a^	ε4 Negative			ε4 Positive		
	B	95% CI	*p*-value	B	95% CI	*p*-value
Iba1	0.002	−0.002–0.005	0.398	0.013	0.006–0.020	0.001*
CD68	0.001	−0.002–0.004	0.496	0.002	−0.004–0.007	0.525
**Aβ**_**1–42**_^**b**^	**OR**	**95% CI**	***p*-value**	**OR**	**95% CI**	***p*-value**
Iba1	1.003	0.995–1.011	0.449	0.993	0.979–1.006	0.288
CD68	1.003	0.998–1.009	0.271	0.995	0.987–1.004	0.257
**Aβ**_**1–40**_^**b**^	**OR**	**95% CI**	***p*-value**	**OR**	**95% CI**	***p*-value**
Iba1	1.006	0.998–1.014	0.124	1.012	0.998–1.027	0.093
CD68	1.003	0.998–1.009	0.229	1.005	0.996–1.014	0.251

^a^Multiple linear regressions were used to assess relationship of Iba1 and CD68 with AT8 cellular density adjusting for age, sex, and Aβ_1–42_. ^b^Ordinal logistic regressions were used to assess relationship of Iba1 and CD68 with Aβ adjusting for age and sex OR, odds ratio; CI, confidence interval. *p < 0.05 after Bonferroni correction for multiple comparisons.

### Associations between cytokines and AD pathological markers

We hypothesized that AD-associated cytokines would display no or weak associations with AD pathology in the absence of *APOE ε*4, and positive associations with pathology in *APOE ε*4 positive participants who are more likely to develop significant AD pathology. Within *APOE* ε4 negative participants, ordinal logistic regression analysis showed a negative association of Aβ_1–42_ with TNF-α (OR = 0.115, *p* = 0.030) controlling for age and sex (Table 4). However, within *APOE* ε4 positive participants, an ordinal logistic regression showed a positive association of Aβ_1–42_ with IFN-γ (*p* = 0.014), IL-10 (*p* = 0.012), and IL-4 (*p* = 0.005) adjusting for age and sex. The association of Aβ_1–42_ with IL-4 in the presence of *APOE* ε4 was still significant after a Bonferroni correction for multiple comparisons. Because high levels of TNF-α are associated with less Aβ_1–42_ in *APOE* ε4 negative participants, TNF-α may have a protective effect on Aβ accumulation that is lost with *APOE* ε4. In contrast, high levels of IL-10, IL-4, and IFN-γ were associated with increased Aβ_1–42_, suggesting an altered cytokine response in the presence of *APOE* ε4. No cytokines displayed a significant association with Aβ_1–40_.

**Table 4:** Cytokine associations with Aβ_1–42_ pathology in *APOE* ε4 negative and positive participants.

	ε4 Negative			ε4 Positive		
	OR	95% CI	*p*-value	OR	95% CI	*p*-value
IL-10	0.880	0.00897–96.2	0.956	1.74 × 10^4^	8.935–3.39 × 10^7^	**0.012**
IL-13	1.001	0.770–1.30	0.995	0.923	0.424–2.010	0.841
IL-4	6.79	0.703–65.8	0.098	6.43 × 10^3^	13.21–3.13 × 10^6^	0.005*
TNF-α	0.115	0.0162–0.815	**0.030**	0.0264	2.23 × 10^-4^–3.13	0.136
IL-1β	0.965	0.920–1.01	0.131	1.066	0.899–1.26	0.462
IL-1α	0.839	0.613–1.23	0.608	1.655	0.586–4.67	0.342
IFN-γ	1.59	0.803–1.60	0.182	5.45	0.385–6.75	**0.014**

Ordinal logistic regressions were run adjusting for age and sex; *p < 0.05 after Bonferroni correction for multiple comparisons; OR, odds ratio; CI, confidence interval.

To test the hypothesis that cytokine associations with tau pathology were altered by the presence of *APOE* ε4, we performed multiple linear regression analyses adjusting for age, sex, and Aβ_1–42_ (Table 5). In *APOE* ε4 negative participants, IL-10 (*p* = 0.015), IL-13 (*p* = 0.003), IL-4 (*p* = 0.021), and IL-1α (*p* = 0.005) were all significantly negatively associated with AT8 tau pathology (Table 5). The negative associations of AT8 tau pathology with increased IL-13 and IL-1α remained significant after a Bonferroni correction for multiple comparisons. In *APOE* ε4 positive participants, there were no significant associations of cytokines with AT8. The negative association of anti-inflammatory cytokines (*i.e*. IL-10, IL-13, IL-4) and IL-1α with tau pathology that is lost in *APOE* ε4 carriers suggests an altered interaction of the brain’s immune system with pathology based on *APOE* genotype.

**Table 5:** Cytokine associations with tau pathology in *APOE* ε4 negative and positive participants.

	ε4 Negative			ε4 Positive		
	B	95% CI	*p*-value	B	95% CI	*p*-value
IL-10	−1.463	−2.634–−0.292	**0.015**	−0.290	−2.826–2.246	0.817
IL-13	−1.687	−2.780–−0.594	0.003*	−2.530	−6.324–1.264	0.183
IL-4	−0.901	−1.662–−0.140	**0.021**	−1.612	−3.912–0.688	0.163
TNF-α	−0.174	−1.099–0.751	0.710	−0.075	−3.076–2.926	0.960
IL-1β	−0.257	−0.679–0.165	0.231	0.548	−0.657–0.433	0.360
IL-1α	−0.906	−1.535–−0.276	0.005*	0.578	−1.491–2.643	0.573
IFN-γ	−0.741	−1.980–0.498	0.223	−2.125	−5.613–1.362	0.223

Multiple linear regressions were run adjusting for age, sex, and Aβ1–42; *p < 0.05 after Bonferroni correction for multiple comparisons. CI, confidence interval. Log transformed values were used when needed to ensure normality. Cytokine concentration in pg/mL.

### Associations between cytokines and microglia cell density

In order to test the association between microglia and those cytokines related to tau pathology (unadjusted *p* < 0.05: IL-10, IL-13, IL-4, and IL-1α), linear regressions were performed adjusting for age and sex (Table 6). Iba1 microglia density was negatively associated with IL-4 in *APOE* ε4 positive participants (*p* = 0.040) and trended towards significance in *APOE* ε4 negative participants (*p* = 0.060). In *APOE* ε4 positive participants there was a trend towards a negative associate with Iba1 density and IL-10 (*p* = 0.055). Conversely, IL-lα exhibited a significant positive association with Iba1 microglia density in *APOE* ε4 positive participants (*p* = 0.023). None of these relationships survived Bonferroni correction for multiple comparisons. IL-13 did not display any significant association to Iba1 microglia density. Overall, the level of the anti-inflammatory cytokine IL-4 was negatively associated with microglia density and the pro-inflammatory cytokine IL-1α was associated with increased microglia density in *APOE* ε4 positive participants.

**Table 6:** Cytokine associations with total microglial density (Iba1) in *APOE* ε4 negative and positive groups.

	ε4 Negative			ε4 Positive		
	B	95% CI	*p*-value	B	95% CI	*p*-value
IL-10	−14.76	−73.91–44.39	0.622	−88.02	−177.87–1.83	0.055
IL-13	6.375	−49.65–62.40	0.822	−37.96	−203.77–127.86	0.644
IL-4	−34.56	−71.88–2.77	0.060	−89.86	−175.386–−4.384	**0.040**
IL-1α	−18.62	−50.32–13.28	0.251	94.42	14.032–174.807	**0.023**

Multiple linear regressions were run adjusting for age and sex. Log transformed values were used when needed to ensure normality. Cytokine concentration in pg/mL.

### Relationship with dementia

To determine whether the two significant cytokines related to tau pathology (*i.e*. IL-13 and IL-1α) had an effect on dementia independent of tau pathology, we performed a logistic regression adjusting for tau pathology, Aβ_1–42_, Iba1, microglia density, age, and sex in *APOE* ε4 positive and negative groups. Only tau pathology was significantly associated with dementia (Table 7). However frontal cortex tau pathology displayed a greater effect on dementia in *APOE* ε4 positive participants (OR = 12.188, *p* = 0.038) than in *APOE* ε4 negative participants (OR = 2.542, *p* = 0.001).

**Table 7:** Associations with dementia in *APOE* ε4 negative and positive participants.

	ε4 Negative		ε4 Positive	
	OR	*p*-value	OR	*p*-value
AT8	2.518	0.001*	12.188	**0.038**
Aβ_1–42_	1.078	0.701	0.717	0.534
Iba1	1.002	0.766	1.003	0.836
IL-13	13.314	0.124	972.365	0.293
IL-1α	0.396	0.337	0.049	0.338

Binary logistic regression adjusting for age and sex; *p < 0.05 after Bonferroni correction for multiple comparisons. Cytokine concentration in pg/mL.

### Sensitivity analyses

In order to control for possible confounds additional analyses were performed adjusting for comorbid pathologies, RNA integrity number (RIN, a measure of tissue quality), and CERAD score. Effect sizes were not changed with adjustments for comorbid pathologies, including arteriolosclerosis, atherosclerosis, and neocortical Lewy bodies or with adjustment for RIN. Adjusting for CERAD score did not significantly alter the association between Iba1 microglial density and AT8 tau pathology, suggesting the relationship is independent of Aβ plaque score.

## Discussion

We examined the pathological relationships between microglia, associated cytokines, and AD pathologies within the dorsolateral frontal cortex in a community-based aging cohort of 154 participants from the Framingham Heart Study. Associations between inflammatory markers and AD pathologies were significantly altered by the presence of the *APOE* ε4 allele. In *APOE* ε4 positive participants, IL-4, IL-10, and IFN-γ were associated with increased levels of Aβ_1–42_. In contrast, higher levels of the cytokines IL-10, IL-13, IL-4, and IL-lα were associated with decreased tau pathology only in the absence of *APOE* ε4. Furthermore, higher Iba1 microglia density was associated with increased tau pathology in *APOE* ε4 positive participants. Compared to *APOE* ε4 negative participants, *APOE* ε4 carriers appear to adopt a potentially toxic association between inflammatory markers and Aβ_1–42_ and lose a potentially protective association between anti-inflammatory cytokines and the development of tau pathology.

While the exact role of *APOE* ε4 in the pathogenesis of AD remains unclear, our findings support its role in inflammation-mediated pathology. ApoE4 has been shown to both impair clearance of Aβ and worsen the inflammation caused by amyloid^29,30^. The presence of *APOE* ε4 has also been associated with increased pro-inflammatory cytokines and microglial activation. A recent study using a transgenic tau mouse model found that the presence of *APOE* ε4 increased tau, the reactivity of microglia, and the level of pro-inflammatory cytokines^31^. Pro-inflammatory cytokine release from microglia led to increased astrocyte activation further increasing inflammation. Furthermore, this study found that mice with *APOE* ε4 had increased expression of pro-inflammatory genes and down-regulation of homeostatic genes^31^.

### Implications for amyloid pathology

**Figure 2:**
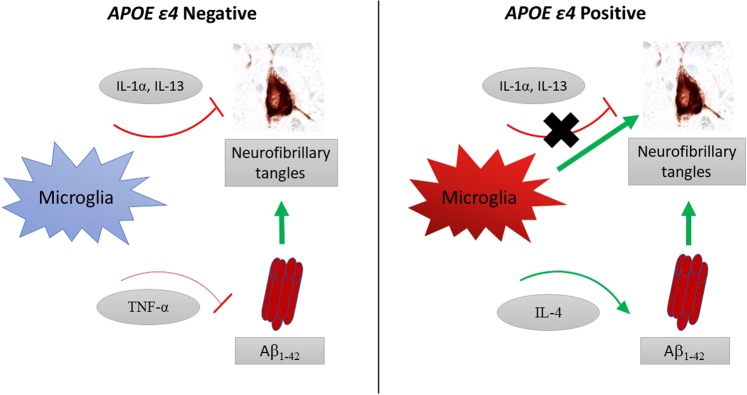
Schematic of significant immune-pathology interactions within *APOE* ε4 positive and negative participants. In *APOE* ε4 negative participants cytokines displayed negative associations with both tau and Aβ_1–42_, and microglia were not associated with neurofibrillary tangles. In contrast, in *APOE* ε4 carriers the negative association of cytokines with tau pathology was lost; microglia density was associated with more neurofibrillary tangles; and IL-4 was associated with increased Aβ_1–42_. Associations with p < 0.05 shown.

The role of microglia on the development of Aβ pathology is complex and varies depending on disease state and experimental paradigm. Our results support previous work in mouse models of Aβ pathology that demonstrated TNF-α decreased Aβ_1–42_ burden^19^ and further suggests that this potential protective effect of TNF-α is present only in *APOE* ε4 negative participants. In contrast, *APOE* ε4 positive participants appear to gain a positive association between increased levels of cytokines IFN-γ, IL-10, and IL-4 with Aβ_1–42_ (Table 3). It is important to note that the very large odds ratio for IL-10 and IL-4 are likely due to the low level of these cytokines in the brain. The cytokines IFN-γ, IL-10, and IL-4 have been proposed to increase amyloid deposition in mouse models of Aβ pathology, which is supported by our findings; however, they have also been associated with decreasing Aβ levels^11,20,32,33^. A study using APP transgenic mice found that increased IL-10 expression was associated with increased amyloid accumulation and increased ApoE expression, particularly within insoluble amyloid plaques^20^. It is proposed that in conjunction with increased IL-10, increased ApoE in these mice significantly impairs the phagocytic ability of microglia leading to the elevated amyloid accumulation. Our results agree with these findings suggesting a detrimental effect of IL-10, IL-4, and IFN-γ on amyloid, particularly in the presence of ApoE4. The difference between cytokine interactions with Aβ_1–42_ in *APOE* ε4 positive and negative participants may reflect an immune state that has an altered and detrimental function in the presence of *APOE* ε4 (Fig. 2).

### Implications for tau pathology

Microglia have been proposed to worsen tau pathology and to be toxic to neurons^34–36^. The reciprocal relationship, that tau induces microglial activation, has also been suggested based on findings in mice that expression of tau increases Iba1 microglia density and also alters the expression of genes involved in inflammation^37,38^. Simultaneous linear regression modeling supports the presence of a positive feedback loop between activated microglia cell density and tau pathology in human brain tissue^27^. We found a significant association between microglia density and tau pathology, suggesting a dysfunctional role of microglia in *APOE* ε4 positive, but not *APOE* ε4 negative, individuals.

A previous post-mortem human study examining the middle frontal cortex found that the association of Iba1 microglia density with tau pathology varied between individuals with and without dementia. In those with dementia Iba1 microglia density was positively associated with tau pathology but displayed a negative association in those without dementia^7^. Here we found that Iba1 microglia density was associated with increasing tau pathology in *APOE* ε4 carriers (Fig. 1), suggesting that an association with dementia may be due to *APOE* ε4 carrier status. Further studies with larger sample sizes are needed to help determine the impact of ApoE on the interaction of microglia with pathology.

Microglia may have a variety of phenotypes that represent different activation states and functions. CD68 is present within lysosomes which are increased in activated microglia with increased phagocytic activity^7,39^. Surprisingly, we found CD68 cell density was not significantly related with any markers of AD pathology. That total microglia density, but not CD68 positive microglial density, is associated with worse tau pathology suggests alternative phenotypes of activated microglia, or potentially astrocytes, may be involved in AD. This disconnect between Iba1 and CD68 positive microglia is supported by Minett *et al*. who found no association between CD68 and Iba1 labelled microglia, while finding weak relationships between other microglia markers – HLA-DR, MSR-A, and CD64 – that were also associated with AD pathology^7^. Future studies should further delineate microglia phenotypes and potential phagocytic or lysosomal impairments involved in human AD pathology.

The relationship between microglia and tau pathology has also been reported in transgenic mouse studies. A mouse line expressing tau and human *APOE* demonstrated that expression of *APOE* ε4 was associated with increased tau phosphorylation^40^. Furthermore, microglia mediated cell damage was shown to be greater in *APOE* ε4 expressing mice than those expressing *APOE* ε3 or *APOE* ε2^41,42^. We found that the association of microglia and associated cytokines with tau pathology is altered by the presence of *APOE* ε4 in human frontal cortex as well.

The detrimental effect of microglia in the presence of *APOE* ε4 may be due in part to the loss of potentially protective cytokine interactions and their decreased levels in *APOE* ε4 positive participants (Table 2). Increasing levels of IL-10, IL-13, IL-4, and IL-1α were associated with decreased tau pathology in *APOE* ε4 negative participants while this effect is absent in *APOE* ε4 carriers (Table 5). The negative association of anti-inflammatory cytokines with tau pathology in *APOE* ε4 negative participants agrees with basic research studies in human cell lines that demonstrate a potentially protective effect^43^.

A variety of proinflammatory cytokines, including IL-1α, have been implicated in AD. IL-1α in its membrane bound form has been observed on activated microglia colocalized with Aβ plaques and neurons laden with tau pathology^44,45^. Additionally, in cultured human astrocytes IL-1α was found to induce tau phosphorylation^46^. Here we found that increased IL-1α was significantly associated with decreased tau pathology independent of Aβ_1–42_ levels within *APOE* ε4 negative participants and that increasing IL-1α levels were associated with increased Iba1 microglia density within *APOE* ε4 positive, but not *APOE* ε4 negative participants. Thus, the interaction between IL-1α and tau pathology appears to be altered by *APOE* ε4.

Tau pathology is one of the major pathological drivers of dementia in aging cohorts^47–49^. The cytokines IL-13 and IL-1α were significantly associated with less tau pathology in *APOE* ε4 negative participants and were not independently predictive of dementia. Instead, tau pathology density in the frontal cortex was significantly associated with dementia and had a much greater effect in *APOE* e4 positive than negative participants. Taken together this might suggest that IL-13 and IL-1α lessen the accumulation and detrimental effect of tau pathology in *APOE* ε4 negative participants and that this protective effect is lost in the presence of *APOE* ε4 (Fig. 2).

Tau pathology is strongly associated with brain atrophy and worsening AD^50–52^. Additionally, tau pathology has previously been shown to correlate with decreased brain decreased brain volumes specifically within the FHS cohort^53^. While our study focuses on the dorsolateral frontal cortex, AD pathology involves several brain regions and appears to spare others^54,55^. In aging and AD, NFTs likely begin within the entorhinal cortex and then extend to limbic areas and into the neocortex, while the opposite has been observed for Aβ pathology, which may begin in the neocortex and then extend to subcortical regions^24,55^. Here we modeled the dorsolateral frontal cortex which is a region where Aβ and tau pathologies converge in intermediate to severe AD. The associations here likely reflect other cortical regions, including visual association areas that have been previously implicated in early AD in this cohort^56^. In addition, cortical layers are differentially affected by pathology such that NFTs in AD tend to involve the deep cortical layers while the plaque pathology may begin more superficially. The patterns of gliosis and regional production of cytokines within different cortical layers requires further study. Further work is needed to determine the progression of pathology in different brain regions over the course of AD.

## Limitations

There were several limitations to this study. Although the FHS brain donation cohort was recruited from the larger FHS community-based study, there is likely an autopsy-based selection bias. Cytokines may be rapidly metabolized and influenced by post-mortem interval. However, adjustment for RIN, a marker of tissue degradation, showed similar results. The associations between numerous cytokines and cell types and pathologies were studied based on *a priori* hypotheses. Nevertheless, future studies will need to confirm these results as well as determine the role of additional cytokines and cell types.

## Conclusions

The associations between microglia and related cytokines with AD pathologies in human frontal cortex were altered between *APOE* ε4 positive and negative participants in a community aging cohort. Increased levels of IL-10, IL-13, IL-4, and IL-1α were associated with lower levels of tau pathology in *APOE* ε4 negative participants, suggesting a potential protective role of the immune system in absence of *APOE* ε4. The negative association of both anti-inflammatory and pro-inflammatory cytokines with tau pathology is absent in *APOE* ε4 positive individuals, who accumulate increased levels of microglia and tau pathology. Overall, *APOE* ε4 appears to alter the associations of microglia and inflammatory cytokines with AD pathologies. This altered immune response may partially explain the more severe Aβ and tau pathology and increased risk for AD within *APOE* ε4 carriers. These findings provide support for a modulatory role of *APOE* in immune and glial cell function in the human brain and highlight the need to stratify by *APOE* genotype when studying immune interactions in AD.

## Data Availability

All materials, data, and associated protocols will be made available.
